# Modulation of sepsis by *Lacticaseibacillus rhamnosus* and the potential role of short-chain fatty acid levels in feces and blood

**DOI:** 10.1038/s41598-025-33032-4

**Published:** 2025-12-24

**Authors:** Wiwat Chancharoenthana, Supitcha Kamolratanakul, Chalisa Pinitchun, Akira Vorapreechapanich, Dhammika Leshan Wannigama, Naraporn Somboonna, Thanya Cheibchalard, Sarn Settachaimongkon, Marcus J Schultz, Asada Leelahavanichkul

**Affiliations:** 1 Department of Clinical Tropical Medicine, Faculty of Tropical Medicine, Mahidol University, 16/F Ratchanakarin Building 420/6 Rajvithi Rd, Ratchathewi, Bangkok, 10400 Thailand; 2 Tropical Immunology and Translational Research Unit (TITRU), Department of Clinical Tropical Medicine, Faculty of Tropical Medicine, Mahidol University, Bangkok, Thailand; 3 Department of Microbiology, Faculty of Medicine, Chulalongkorn University, Bangkok, Thailand; 4 Center of Excellence on Translational Research in Inflammation and Immunology (CETRII), Department of Microbiology, Faculty of Medicine, Chulalongkorn University, Bangkok, Thailand; 5 Department of Infectious Diseases, Faculty of Medicine, Yamagata University, Yamagata University Hospital, Yamagata, Japan; 6 Department of Infectious Diseases and Infection Control, Yamagata Prefectural Central Hospital, Yamagata, Japan; 7 Pathogen Hunter’s Research Collaborative Team, Department of Infectious Diseases and Infection Control, Yamagata Prefectural Central Hospital, Yamagata, Japan; 8 Yamagata Prefectural University of Health Sciences, Kamiyanagi, Yamagata 990-2212 Japan; 9 Biofilms and Antimicrobial Resistance Consortium of ODA receiving countries, The University of Sheffield, Sheffield, UK; 10 Department of Microbiology, Faculty of Science, Chulalongkorn University, Bangkok, Thailand; 11 Microbiome Research Unit for Probiotics in Food and Cosmetics, Chulalongkorn University, Bangkok, Thailand; 12 Program in Biotechnology, Faculty of Science, Chulalongkorn University, Bangkok, Thailand; 13 Department of Food Technology, Faculty of Science, Chulalongkorn University, Bangkok, Thailand; 14 Department of Intensive Care & Laboratory of Experimental Intensive Care and Anesthesiology (L.E.I.C.A), Academic Medical Center, University of Amsterdam, Amsterdam, The Netherlands; 15 Centre for Tropical Medicine and Global Health, Nuffield Department of Medicine, Oxford University, Oxford, UK; 16 Mahidol–Oxford Tropical Medicine Research Unit, Faculty of Tropical Medicine, Mahidol University, Bangkok, 10400 Thailand; 17 Immunology Unit, Department of Microbiology, Chulalongkorn University, Bangkok, 10330 Thailand

**Keywords:** Metabolome, Short-chain fatty acids (SCFAs), Sepsis, Enterocytes, Mice, Cell biology, Immunology, Microbiology, Molecular biology

## Abstract

The efficacy of probiotics for sepsis attenuation might be associated with the alteration of short-chain fatty acids (SCFAs). We investigated the impact of probiotics with the different production of SCFAs in vitro, including *Lacticaseibacillus rhamnosus* strains fa1 and fg2 in mice with cecal ligation and puncture. Administration of either fa1 or fg2 probiotics, but not the heat-killed probiotics, prior to surgery effectively reduced sepsis severity. Metabolome analysis revealed elevated levels of acetate and 3-hydroxybutyrate in blood, whereas butyrate and propionate levels were diminished in the feces of sepsis mice compared to sham controls. Both probiotics similarly attenuated sepsis-induced gut dysbiosis, as indicated by the normalized Firmicutes and reduced Proteobacteria (fecal microbiome analysis), with the similar levels of fecal SCFAs. In parallel, the administration of butyrate, but not acetate, partly attenuated sepsis severity (gut permeability and serum TNF-α). Conditioned media from both probiotic strains or butyrate demonstrated a protective effect against enterocyte injury following activation by *Klebsiella pneumoniae* lysate, irrespective of their SCFAs production. To support the possible use of SCFAs in sepsis, the lower serum SCFAs in patients with sepsis compared to healthy controls was demonstrated. In conclusion, both fa1 and fg2 attenuated sepsis severity, partly through the increased levels of SCFAs. These findings endorse the potential of probiotics in preventing sepsis and the use of SCFAs for sepsis disease monitoring.

**Supplementary Information:**

The online version contains supplementary material available at 10.1038/s41598-025-33032-4.

## Introduction

Mortality rates of sepsis, a common syndrome in response to severe infection, remain unacceptably high^1,2^. The innate and adaptive immunity are important targets in sepsis^3^, but interventions remained largely unsuccessful. Sepsis also induces important changes in the metabolome^4,5^, partly due to rapid breakdown of proteins, carbohydrates, and fats^6,7^, with alterations in the connection between the intestine and the blood^8–10^. Because of the massive alteration of metabolic products in sepsis, the metabolome analysis receives increasing attention as another potential target in sepsis research. Few studies, however, analyzed blood and fecal content metabolomes simultaneously. As metabolomic changes in the blood and the fecal contents during sepsis may differ, simultaneous exploration of blood and fecal metabolomes from a sepsis animal model (which has fewer confounding factors than patients with sepsis) may be warranted.

Short-chain fatty acids (SCFAs), including acetate, propionate, and butyrate, are metabolic products of anaerobic bacterial fermentation in the intestine, especially in the context of complex carbohydrate-rich diets, are important for maintenance of the intestinal gut barrier^11^. Due to sepsis-induced changes in gut microbiota, particularly reduction of Firmicutes, the major SCFAs-producing bacteria^8,9,12,13^, SCFAs depletion can occur during sepsis and administration of butyrate has been proposed as a sepsis attenuation strategy^14^. SCFAs are the main metabolites produced in the colon by the fermentation of dietary fibers and resistant starch^15^ and are easily absorbed by enterocytes^16,17^. Administration of probiotics, particularly those that produce SCFAs, may attenuate sepsis severity. As such, *Lacticaseibacillus rhamnosus* species (previously known as *Lactobacillus rhamnosus*) are lactic acid producing bacteria that are often used among several available strains of probiotics because of their tolerance to acidity (stomach) and alkalinity (intestinal bile) with the relatively easy preparation procedure^18^.

We tested the effects of probiotics using strains with different properties in the production of SCFAs using the isolated strains from the healthy Thai volunteers in our recent study^18^. In parallel, a commercially available butyrate was also used in mice subjected to cecal ligation and puncture (CLP) to generate sepsis^19,20^. Metabolome analysis of blood and fecal content was performed to explore SCFAs in the model. We hypothesized that sepsis severity reduces the abundance of SCFAs, and the probiotics, as well as butyrate, attenuates sepsis-induced intestinal damage.

## Materials and methods

### Animals and animal model

C57BL/6 mice (8 weeks old) were purchased from Nomura Siam International (Pathumwan, Bangkok, Thailand). The study protocol was approved by the Institutional Animal Care and Use Committee of the Faculty of Medicine, Chulalongkorn University, Bangkok, Thailand, following the National Institutes of Health, USA (the ethical approval number: ASP SST 015/2565) in accordance with the Animal Research: Reporting of In Vivo Experiments (ARRIVE) guidelines and regulations. The cecal ligation and puncture (CLP) surgery was performed to induce sepsis, while the sham operation was conducted as control. For probiotic treatment, *L. rhamnosus* strains fa1 and fg2 (Chulalongkorn University, Bangkok, Thailand), which were isolated from Thai healthy volunteers in a previous study^18^, at 1 × 10^8^ CFU in 0.3 mL PBS, or PBS alone (vehicle group), were orally administered daily for 7 days prior to CLP or sham operation. Additionally, the heat-killed fa1 and fg2 were also tested in sepsis. The heat-killed probiotics were prepared by immersion in a water-bath at 60 °C for 1 h as previously described^21,22^. Because butyrate and acetate, the SCFAs, produced from the probiotics might be, at least in part, responsible for sepsis attenuation, both butyrate and acetate were also tested in CLP sepsis mice following previous publications^14,23,24^. As such, sodium butyrate or sodium acetate (Sigma-Aldrich, St. Louis, MO, USA) at 500 mg/kg bodyweight (0.3 mL) or PBS was orally administered at 0.5 and 4 h after CLP. For CLP and sham surgery, all surgical processes were performed under isoflurane anesthesia, following a previous protocol^14,22,25,26^. Briefly, a midline abdominal incision was created, the cecum ligated at 1 cm from the cecal tip, punctured twice with a 21-gauge needle, and gently squeezed to express a small amount of fecal material, before closing the abdominal wall layer by layer with Nylon 4 − 0 sutures. Then, tramadol (25 mg/kg/dose) in 0.25 mL prewarmed normal saline solution (NSS) and imipenem/cilastatin (14 mg/kg/dose) in 0.2 mL NSS were subcutaneously administered in both flanks after surgery, and at 6 and 18 h post-CLP. In the sham operation, the cecum was simply isolated and the abdomen closed by suturing. At 24 h after sham (control) or CLP surgery, mice were euthanised by isoflurane anesthesia before the collection of (i) blood through cardiac puncture procedure and (ii) fecal samples (feces from all parts of the colon were combined and used for fecal metabolome analysis).

## Mouse sample analysis and gut permeability test

For gut permeability analysis, the detection of FITC–dextran (molecular weight 4.4 kDa) (Sigma-Aldrich), which is a non-intestinal absorbable molecule, in serum after an oral administration represents gut permeability defect^27^. As such, FITC–dextran (0.5 mL) at a concentration of 25 mg/ 1 mL PBS before being measured FITC-dextran in serum at 3 h later using a fluorospectrometry (NanoDrop 3300; Thermo Scientific, Wilmington, DE, USA). In the time-point experiment using FITC-dextran, different mice for each time-point were used because of the retention of FICT-dextran in the mouse’s gut for a few days after an oral administration^28^. Kidney function was determined by analysis of blood urea nitrogen and serum creatinine using QuantiChrom assays for urea (DIUR-100) and creatinine (DICT-500) (BioAssay, Hayward, CA, USA). Serum alanine transaminase liver enzyme function was measured by EnzyChrom Alanine Transaminase assay (EALT-100) (BioAssay). The evaluation of kidney and lung histology on 10% formalin fixed with paraffin-embedded slides stained by hematoxylin and eosin (H&E) color (kidneys and lungs) and Masson trichrome (Masson) color (kidneys) using 200x magnification in 20 randomly selected fields for each animal was performed. The semi-quantitative lung injury score is based on alveolar congestion and neutrophil infiltration using the following score: 0 points, no injury in the observed field; 1 point, injury up to 25%; 2 points, injury up to 50%; 3 points, injury up to 75%; 4 points, injury to the entire field. Renal injury score in H&E stained color was defined by the area of the injury as indicated by tubular epithelial swelling, loss of brush border, vacuolar degeneration, necrotic tubules, cast formation, and desquamation using the following score: 0, area < 5%; 1, area 5–10%; 2, area 10–25%; 3, area 25–50%; 4, area > 50%. Meanwhile, the area of renal fibrosis (blue color) in Masson’s-trichrome-stained sections was determined by the computerized image analysis software (ImageJ^®^ software, Bethesda, MD, USA) in a 200× magnification field with 10 fields per sample^29^. Spleen samples (4 mm paraffin sections) were also stained using hematoxylin and eosin and anti-activated caspase-3 immunohistochemistry (Cell Signaling, Technology, Beverly, MA, USA). Caspase-3 positive cells per high-power field, evaluated in 10 randomly selected ×200 magnified fields per slide, were considered to represent apoptotic cell abundance in the spleen, following previous publications^30–32^. Additionally, colon samples (representative of the intestine) in Cryogel (Leica Biosystems, Richmond, IL, USA) were cut into 5-µm-thick frozen sections, fixed in acetone, blocked using blocking buffer, and stained with a fluorescent antibody against occludin-1, claudin-1, and zona occludens-1 (ZO-1) with an Alexa Fluor 488-conjugated (green) secondary antibody (Life Technologies, Carlsbad, CA, USA) before visualization and scoring under a ZEISS LSM 800 confocal microscope (Carl Zeiss). To evaluate the abundance of tight junction proteins (occluding-1 and ZO-1) in the colon tissue, the ascending colon at 2 cm next to the cecum was collected and determined with quantitative reverse transcription polymerase chain reaction (qRT-PCR). Briefly, the colon samples were cleaned with NSS, weighed, and the total RNA from the samples was prepared using an RNA-easy mini kit (Qiagen, Hilden, Germany) and quantified by a Nanodrop 1000 Spectrophotometer (Thermo Scientific). Then, the total reverse transcribed RNAs were processed with a High-Capacity cDNA Reverse Transcription before performing qRT-PCR with SYBR Green PCR Master Mix using QuantStudio6 Flex Real-time PCR System (Thermo Scientific). The lists of primers were; occludin (*OCLN*), forward 5’-CCAATGTCGAGGAGTGGG-3’, reverse 5’-CGCTGCTGTAACGAGGCT-3’; Zona-occludens-1 (*ZO-1*), forward; 5’- GCAAGAGGAGTCCCTGACTG-3’, reverse; 5’-CGGCTCTGTCCTAACTCCAG-3’, and *GAPDH*, forward 5’-GCACCGTCAAGGCTGAGAAC-3’, reverse 5’-ATGGTGGTGAAGACGCCAGT-3’. Additionally, serial dilutions of mouse blood samples were directly spread onto blood agar plates (Oxoid, Hampshire, UK), incubated at 37 °C for 24 h before colony enumeration to measure bacterial abundance (bacteremia) and cell-free DNA evaluation by Quanti PicoGreen assay (Sigma-Aldrich). Serum lipopolysaccharide (LPS) and cytokines (TNF-α, IL-6, and IL-10) were evaluated by HEK-Blue LPS detection (InvivoGen, San Diego, CA, USA) and enzyme-linked immunosorbent assay (ELISA) (Invitrogen, Waltham, MA, USA), respectively.

## Metabolome analysis of blood and fecal samples

Following earlier studies, samples were prepared, and metabolome analysis was conducted using nuclear magnetic resonance (NMR) spectroscopy^33^. Briefly, plasma (400 µL) or feces (0.2 g) at pH 7.5 in 2.4 mL ultrapure water was vortexed for 10 min, and centrifuged (14,000 × g, 10 min, 4 °C) before transferring supernatants (500 µL) into Eppendorf tubes for filtering through a Pall Nanosep^®^ (3 kDa molecular weight) filter (Pall Life Science, Ann Arbor, MI, USA). Buffer (300 mM KH_2_PO_4_, 10% (w/w) deuterium oxide (D_2_O), and 1 mM 3(trimethylsilyl) propionic acid sodium salt (TSP), pH 7.5) was then combined 1:1 (vol: vol) with the filtrate. Then, 1 H NMR spectra were aligned and calibrated using the internal standard (TSP) peak in a 500 MHz NMR spectrometer (Bruker, Rheinstetten, Germany); nuclear overhauser enhancement spectroscopy 1D1HNMR experiments were also conducted. Chemical shift (d) in the range 0.00–10.00 ppm was segmented (binned) at 0.02 ppm intervals for each spectrum, and Topspin (V 4.0.7, Bruker Biospin) used to integrate the signal intensity in each bin to determine the quantity of each spectrum. Metabolite identification for each spectrum was in accordance with the ChenomxNMR suite 8.5 library (Chenomx Inc., Alberta, Canada). MetaboAnalyst 5.0 (http://www.metaboanalyst.ca/) (sample median and auto-scaled mean centering and division by the standard deviation of each variable), clustering algorithm by Ward’s method, and statistical analyses were applied for metabolome data normalization. Data were visualized using GraphPad Prism version 8.0 software (GraphPad, La Jolla, CA, USA) and MetaboAnalyst 5.0. All animal experiments included clearly defined biological replicates. Specifically, the intervention experiments (probiotic administration and short-chain fatty acids (SCFAs) treatments) consistently used between 5 and 7 individual mice per experimental group. For metabolomics analysis, blood samples included 3–4 biological replicates per group, whereas fecal metabolomic analyses comprised 5 biological replicates per group.

## SCFAs evaluation

SCFAs in *L. rhamnosus* culture supernatants were evaluated by gas chromatography–mass spectrometry (GC-MS) using the headspace solid-phase microextraction method with an Agilent 6890 GC equipped with an Agilent 5973 mass selective detector (Agilent Technologies), following the previous publications^33–35^. Briefly, for fecal SCFAs, feces (20 mg in 500 µL of normal saline) were added to 10% H_2_SO_4_ before fatty acid separation using anhydrous ether (800 mL) and centrifugation (18,000 × g, 15 min). Next, the upper ether phase was mixed with 0.25 g anhydrous Na_2_SO_4_ (30 min), centrifuged (18,000 × g, 5 min), and SCFAs in the upper diethyl ether phase determined by GC-MS. For serum, 50 µL of the internal standard solution (150 µM acrylic acid with 1,500 µM m-phosphoric acid) was added to 100 µL serum and centrifuged (18,000 × g, 30 min) before solidification of the precipitate for 30 min (4 °C). Then, propyl formate (100 µL) was added in the clear supernatant (100 µL) in a new tube, followed by centrifugation (18,000 × g, 10 min), and 50 µL of the organic layer was placed into gas chromatography (GC) vials (the blank tubes were prepared similarly without an added serum sample). The dimensions of the column and temperature program were 0.25 mm × 30 m × 0.25 mm with helium carrier gas at 13.7 ml/min and 10 min isothermal at 50 °C, 10 min rising to 240 °C at 15 °C/min, respectively. The temperature of the injection and detector ports were 200 °C and 250 °C, respectively. The mass spectrometer was operated in electron impact mode at 70 eV with scan range 40–200 amu. A standard curve was obtained for calculation of each SCFAs concentration.

### Fecal microbiome analysis and fecal abundance of *Lacticaseibacilli* spp.

Fecal microbiome analysis was performed following previous publications^36,37^. Briefly, feces of mice from different cages were collected to avoid the impacts of coprophagy (the consumption of feces from other mice in the same cage). At sacrifice, all fecal samples from the cecum and colon were combined and 0.3 g feces per mouse were processed for metagenomic DNA extractions using the DNeasy Kit (Qiagen GmbH, Hilden, Germany), and DNA quality was assessed by nanodrop spectrophotometry. The universal prokaryotic primers 515F (5’-GTGCCAGCMGCCGCGGTAA-3’) and 806R (5’-GGACTACHVGGGTWTCTAAT-3’), with an appended 5’ Illumina adapter and 3’ Golay barcode sequences, were used for 16 S ribosomal RNA gene V4 library construction. After that, the samples were normalized to an equal sampling depth (*N* = 48,430 reads per sample). The rarefaction curve, Good’s coverage, and alpha diversity indices (Chao richness and Shannon diversity) were computed using the Mothur method. In parallel, the abundance of fecal *Lacticaseibacilli* was also determined by real-time polymerase chain reaction (PCR) according to a previous published protocol^38^. Briefly, the total fecal DNAs were extracted by a QIAamp Fast DNA Stool Mini Kit (Qiagen, Hiden, Germany) following the manufacturer’s instructions with the primers for variable regions of the 16 S rRNA gene sequence of *L. rhamnosus*; rham (forward; 5’-TGCATCTTGATTTAATTTTG-3’) and Y2 (reverse; 5’-CCCACTGCTGCCTCCCGTAGGAGT-3’)^39^. The amplicon was approximately 290 base pairs (bp), and the genome size of *L. rhamnosus* (also designated as LR ATCC 53103) was 3,005,051 bp^40^, while the bacterial genome is approximately 1.98 × 10^9^ g/mol and contains 6.02 × 10^23^ molecules/mol. Additionally, one bacterium corresponds to 3.3 fg of DNA, and the standard curve was generated by the QuantStudio™ Design & Analysis Software v1.4.3 (Thermo Fisher Scientific) using 10-fold serial dilution (6.6 fg to 660 pg) with bacterial concentrations ranging from 2 to 2 × 10^5^ bacteria.

The probiotic dosage selected for this study (1 × 10^8^ CFU per mouse per day) was determined based on previously published animal models demonstrating safety and efficacy in murine sepsis models^33,36^. Translationally, this dosage aligns with probiotic dosages commonly administered to human adults (approximately 1 × 10^9^ to 1 × 10^10^ CFU per day), thereby supporting the potential for clinical relevance^41,42^.

### In vitro enterocyte assay

Given the potential influence of some probiotics-derived molecules on intestinal integrity^43,44^, in vitro experiments using conditioned media from *L. rhamnosus* cultures (LCM) were conducted. Due to enterocyte resistance against LPS, heat-killed *Klebsiella pneumoniae* were used to stimulate the enterocyte cell line. Briefly, human colorectal adenocarcinoma cells (Caco-2, American Type Culture Collection, Manassas, VA, USA; ATCC HTB-37) were maintained in supplemented Dulbecco’s modified Eagle medium (DMEM) and *Klebsiella pneumoniae* ATCC 13,883 were grown on tryptic soy agar (Oxoid, Hampshire, UK) supplemented with 5% sheep blood under aerobic conditions. Bacteria were prepared by incubation at 70 °C for 45 min and sonication for 1 h before application to activate Caco-2 cells. To prepare LCM, fa1 or fg2 (OD600 = 0.1) was incubated anaerobically for 48 h before collection of cell-free supernatant by centrifugation and filtration through 0.22-µm membrane filters (Minisart; Sartorius Stedim Biotech GmbH, Göttingen, Germany). Then, 500 µl of the preparation was concentrated by speed vacuum drying at 40 °C for 3 h (Savant Instruments, Farmingdale, NY), before resuspension of the cell-free concentrated pellets in an equal volume of DMEM and storage at 20 °C until use. Subsequently, Caco-2 cells (5 × 10^4^ cells/well) were treated with LCM (5%, vol/vol) from fa1 or fg2, together with the heat-killed bacteria (1.5 × 10^7^ CFU/well) under 5% CO_2_ at 37 °C for 24 h. Next, culture supernatants were prepared by centrifugation (125 g, 4°C, 7 min), and levels of TNF-α and IL-8 measured by Quantikine ELISA immunoassay (R&D Systems, Minneapolis, MN, USA). In parallel, sodium butyrate (Sigma-Aldrich) (2 mM) was also tested in Caco-2 cells because of the possible impact of the secreted butyrate in the LCM of the probiotics^45^.

To measure TEER, Caco-2 cells (5 × 10^4^ per well) were seeded into the upper compartments of 24‐well Boyden chamber trans wells (Sigma‐Aldrich) in modified DMEM for 15 days, to establish a monolayer. Cells were then incubated with heat-killed bacteria (1.5 × 10^7^ CFU/well; preparation described above), for 24 h before TEER measurement in ohm (W) × cm^2^, using an epithelial volt‐ohm meter (EVOM2™, World Precision Instruments, Sarasota, FL, USA) by placing electrodes in the supernatant in the basolateral and apical chambers. TEER values in culture media without Caco‐2 cells were used as a blank control and subtracted from all other measurements. In parallel, gene expression levels in enterocytes were assessed by quantitative reverse-transcription polymerase chain reaction (qRT-PCR), as previously described^46,47^. Briefly, total RNA was extracted from treated cells using TRIzol reagent (Invitrogen) and 50 ng RNA converted into complementary DNA using a high capacity reverse transcription assay (Applied Biosystems, Warrington, UK), followed by PCR analysis of gene expression levels relative to those of glyceraldehyde 3-phosphate dehydrogenase (*GAPDH*) using SYBR Green PCR Master Mix and QuantStudio™ Design & Analysis Software v1.4.3 (Thermo Fisher Scientific, Foster City, CA, USA). Expression levels were calculated using the 2^−∆∆Ct^ method. Primers used for nuclear factor-κB (*NF-κB*) were forward 5’-ATGGCTTCTATGAGGCTGAG-3’, reverse 5’-GTTGTTGTTGGTCTGGATGC-3’, while primers for occludin (*OCLN*), Zona-occludens-1 (*ZO-1*)^48,49^, and *GAPDH* were mentioned above. In addition, Western blot analysis was used to determine the tight junction molecules (ZO-1 and occludin) following the previously published protocol^48,49^. In brief, cell lysates in RIPA (Radio-Immuno-Precipitation Assay) buffer supplemented with protease inhibitor cocktail (Thermo Fisher Scientific, Waltham, MA, USA) were prepared and the samples at 20 µg of total protein, as determined by bicinchoninic acid assay (Thermo Fisher Scientific), were subjected to SDS-PAGE, transferred onto PVDF (polyvinylidene difluoride) membranes, blocked by 5% BSA (bovine serum albumin) in TBST (Tris-buffered saline with 0.05% Tween 20) buffer and incubated with specific primary antibodies against ZO-1 (anti-ZO-1 1A12) (Invitrogen), occludin (anti-occludin OC-3F10) (Invitrogen), and GAPDH (anti-GAPDH D16H11) (Cell-Signaling Technology, Beverly, MA, USA) overnight at 4°C. Then, the secondary antibody linked with horseradish peroxidase enzyme was used and visualized by ImageQuant™ LAS 500 (GE-Healthcare, Marlborough, MA, USA).

## Patient’s samples

The Ethics Committees of the King Memorial Chulalongkorn Hospital (KMCH) approved usage of blood samples from the healthy volunteers and patients with sepsis under IRB No. 610/64. All experiments were conducted in accordance with the Declaration of Helsinki guidelines and regulations. Written informed consent was obtained from all participants prior to sample collection. As such, the cross-sectional analysis of the blood samples from patients who were admitted to the intensive care unit (ICU) of KMCH between December 2022 and October 2023 and the healthy volunteers was performed. The inclusion criteria were age > 18 years, diagnosed by at least 2 physicians, and sequential organ failure assessment (SOFA) scores higher than 2, while the exclusion criteria were pregnancy, hematologic diseases, neutropenia, the use of granulocyte-colony stimulating factor and immunosuppressive drugs, and organ transplantation. The initial sepsis severity using the SOFA scores was performed at the enrollment. The routine laboratory results from the serum samples were measured at the central laboratory of the King Chulalongkorn Memorial Hospital, including complete blood count, serum creatinine, and serum alanine transaminase, using the Sysmex XN9203 Analyzer (Kobe, Hyogo, Japan) and Cobas c502 (Roche Diagnostics, Basel, Switzerland). Meanwhile, serum cytokines (TNF-α and IL-6) were measured by enzyme-linked immunosorbent assay (ELISA) (Invitrogen, Waltham, MA, USA).

### Statistical analysis

Mean ± standard error of the mean values is presented and were compared using one-way analysis of variance followed by Tukey’s analysis for multiple group comparison or Student’s t test for comparisons between two groups. Survival was analyzed using the log-rank test. All statistical analyses were performed using Graph Pad Prism version 9.5.1 software (La Jolla, CA, USA) and *p*-value < 0.05 was considered statistically significant. Due to the exploratory nature of the metabolomics analysis, p-values for individual metabolites reported herein were not adjusted for multiple testing. Rather, metabolites showing substantial magnitude of change, statistical significance, and clear biological plausibility were prioritized for reporting. However, we recognize the potential limitations and risk of type I errors. Future confirmatory studies specifically aiming at metabolite biomarker validation should employ rigorous correction methods such as the False Discovery Rate (FDR) or Bonferroni corrections^50^.

## Results

### Blood and fecal metabolome alterations in CLP sepsis mice

**Fig. 1:**
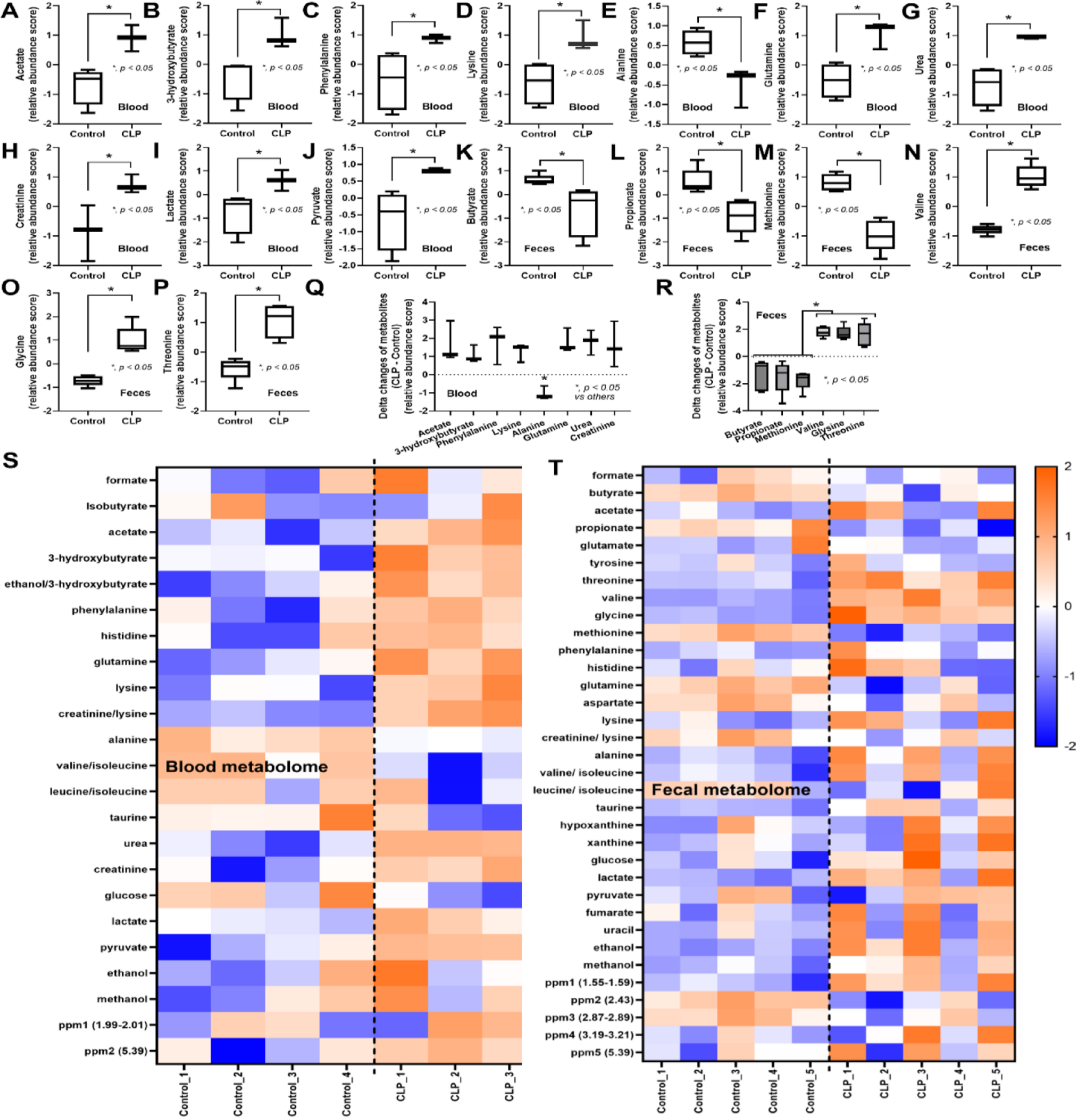
Plots of metabolites that differed between sham (control) versus cecal ligation and puncture (CLP) mice at 24 h post-operation in metabolome analysis of blood (**A**–**J**) (*n* = 4 for control and *n* = 3 for CLP in the blood metabolome) and feces (**K**–**P**) (*n* = 5 per group in the fecal metabolome), the delta changes of the metabolites (the relative abundance in CLP mice – the value of the control group) from blood (**Q**) and feces (**R**), and the heatmap of metabolite data generated by the analysis of metabolomes (the qualitative and quantitative collection of all low-molecular-weight molecules) in blood (**S**) and feces (**T**) (the color scale bar indicates log_2_ fold-change, and the red and blue colors represent high and low levels of the metabolites, respectively) are demonstrated. *p < 0.05 between the indicated groups. The 2 and more than 2 groups comparison were determined by the Student’s t test and one-way analysis of variance followed by Tukey’s analysis.

Because the low molecular weight molecules (metabolites) in blood and serum might be altered after sepsis, metabolome analysis was performed. As such, blood metabolome data indicated that acetate (a SCFAs produced by gut bacteria)^51^ and 3-hydroxy butyrate (an energy source synthesized from the liver)^52^ were elevated in CLP mice (Fig. 1A, B). Additionally, several amino acids (phenylalanine, lysine, and glutamine) (Fig. 1C, D, F), uremic toxins (urea and creatinine) (Fig. 1G, H), and energy-associated molecules (lactate and pyruvate) (Fig. 1I, J) were increased in CLP mice when compared with sham control group. Meanwhile, blood alanine levels in CLP mice were lower than those in controls (Fig. 1E). In the fecal metabolome, the decreased SCFAs (butyrate and propionate) and reduced methionine amino acid, with an elevation in other amino acids (valine, glycine, and threonine) (Fig. 1K–P) were demonstrated, possibly due to sepsis-induced changes in the bacterial microbiota^8^. For a clearer presentation of the alteration of metabolites in blood and feces caused by sepsis, the delta changes of the metabolites (the difference between the relative abundance score of CLP and control) were shown (Fig. 1Q, R). As such, most of the metabolites in the blood of sepsis mice were elevated, except for alanine, which was lower in CLP mice compared with controls (Fig. 1Q). Meanwhile, there was a reduction of fecal SCFAs (butyrate, propionate, and methionine) and an elevation of fecal amino acids (valine, glycine, and threonine) (Fig. 1R). Indeed, the nuclear magnetic resonance (NMR)-based metabolome analysis of blood samples from CLP sepsis mice clearly demonstrated elevated levels of most metabolites relative to sham control mice at 24 h post-surgery (Fig. 1S, red color). Meanwhile, the differences in fecal metabolome profiles between CLP and control group mice were less clear (Fig. 1T). Additionally, the differences in blood and fecal metabolomes between sham control versus CLP sepsis mice were also detected by partial least squares discriminant analysis and principal component analysis (Supplement Fig. 1A–D). Given the reductions in butyrate and propionate detected in the feces of CLP mice, we next investigated the effects of administration of SCFAs-producing probiotics on this sepsis model.

### Sepsis Attenuation and enterocyte protection by probiotic strains with differing SCFAs production properties

**Fig. 2:**
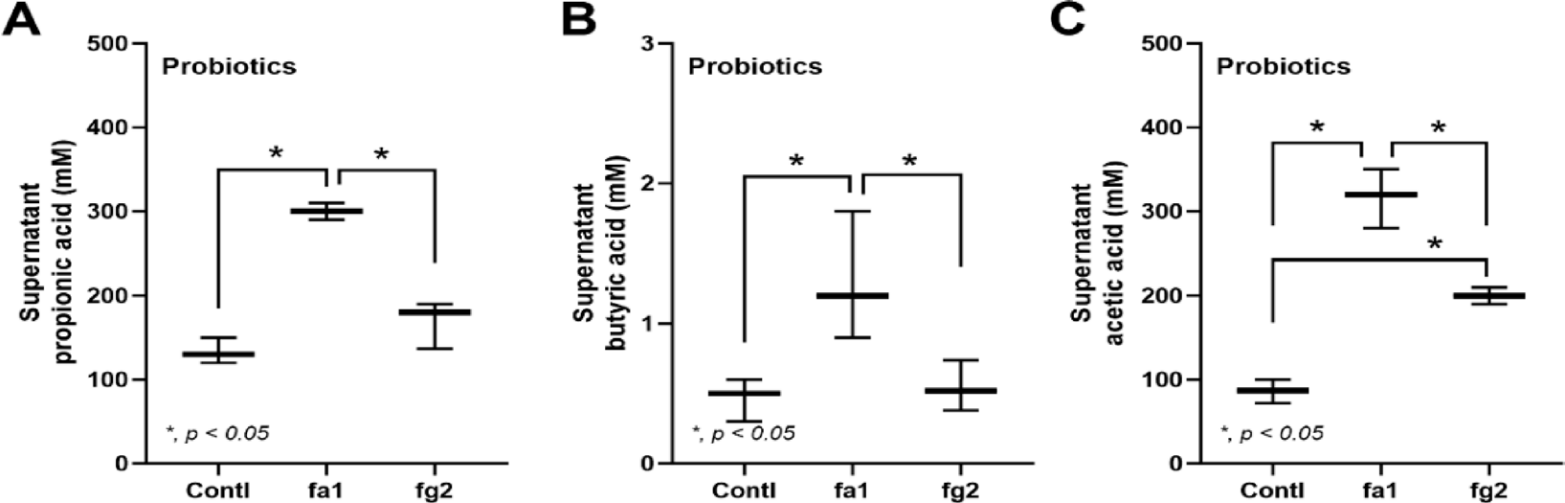
The short-chain fatty acid (SCFAs) (acetic, butyric, and propionic acids) in conditioned media from *L. rhamnosus* fa1 and fg2 probiotic strains compared with those in control media (Contl) (**A**–**C**) (*n* = 5–7 per group) are shown. Independent experiments were performed in triplicate for the in vitro experiments. *p < 0.05 between the indicated groups. The comparison was determined by the Student’s t test and one-way analysis of variance followed by Tukey’s analysis.

**Fig. 3:**
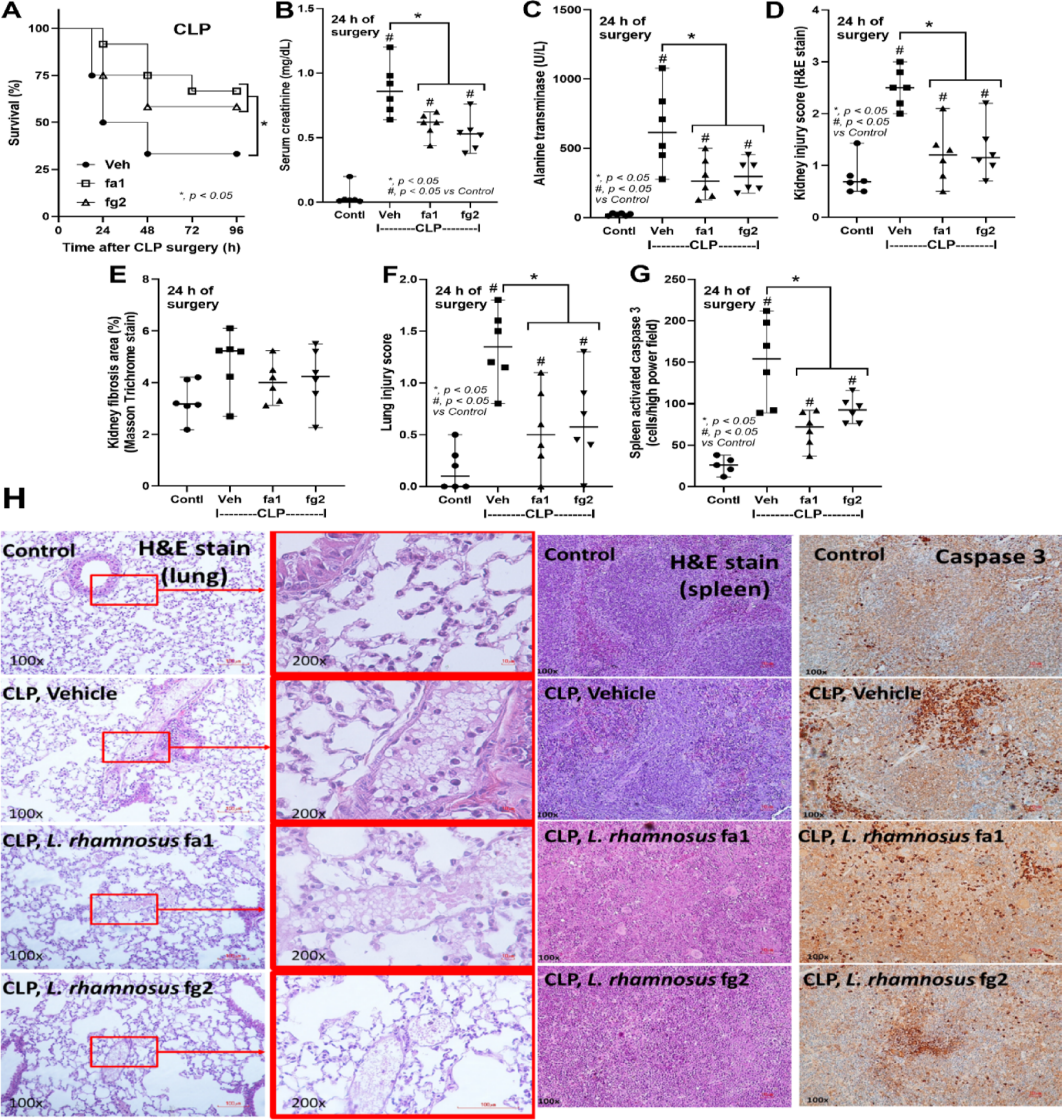
Characteristics of mice in the sham control (Contl) versus cecal ligation and puncture (CLP) groups administered *L. rhamnosus* fa1 (fa1), *L. rhamnosus* fg2 (fg2), or the vehicle control (PBS; Veh) at 24 h post-operation, including: survival (**A**), renal injury (serum creatinine) (**B**), liver damage (serum alanine transaminase) (**C**), kidney histological injury score (H&E stain and Masson’s trichrome) (D, E), lung injury score (F), spleen apoptosis (cells with positive anti-activated caspase 3) (G), the representative H&E stain of lungs with representative immunohistochemistry anti-caspase 3 images in spleens (**H**), are demonstrated (*n* = 6 per group). *p < 0.05 between the indicated groups; ^#^*p* < 0.05 vs. control (Contl). The survival analysis was calculated by the log-rank test, while other comparisons were determined by the Student’s t test and one-way analysis of variance followed by Tukey’s analysis.

**Fig. 4:**
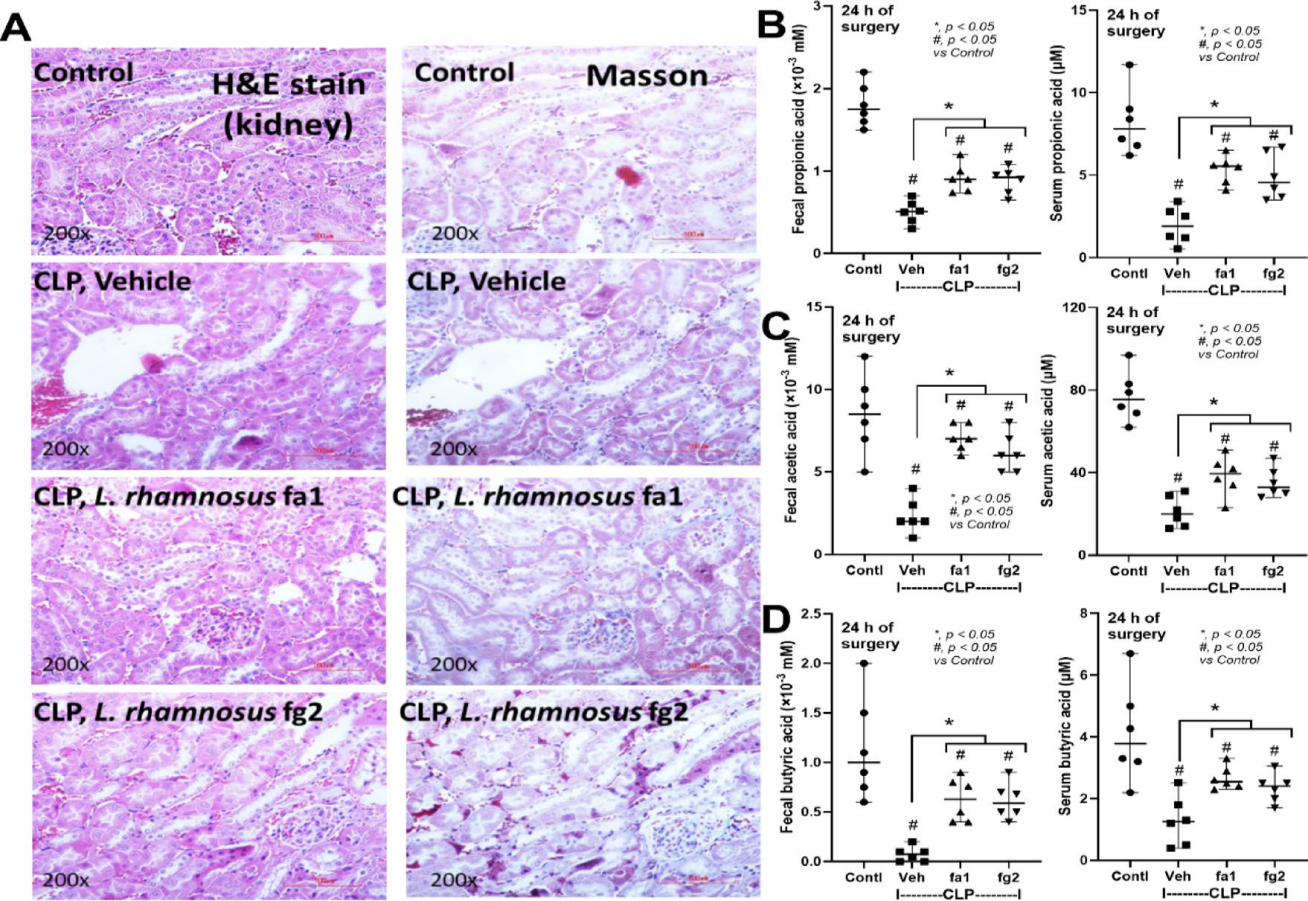
Characteristics of mice in the sham control (Contl) versus cecal ligation and puncture (CLP) groups administered *L. rhamnosus* fa1 (fa1), *L. rhamnosus* fg2 (fg2), or the vehicle control (PBS; Veh) at 24 h post-operation, including: the representative pictures of kidney histology; Hematoxylin and Eosin (H&E) stain (left) and Masson’s trichrome (right) (**A**), and short-chain fatty acid (SCFAs) (acetic, butyric, and propionic acids) in feces and serum (**B**–**D**) (*n* = 6 per group), are demonstrated. *p < 0.05 between the indicated groups; ^#^*p* < 0.05 vs. control (Contl). The comparison was determined by the Student’s t test and one-way analysis of variance followed by Tukey’s analysis.

**Fig. 5:**
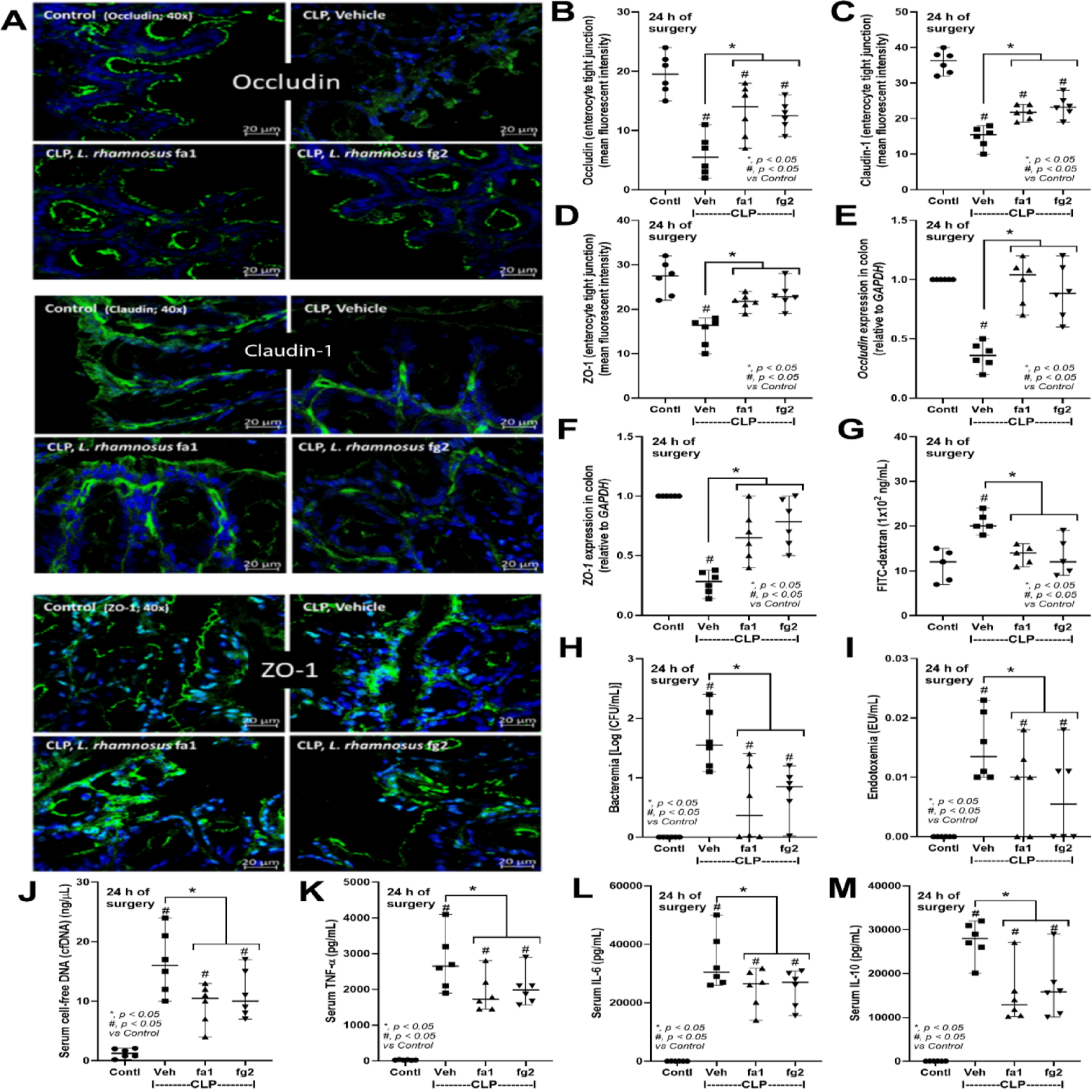
Characteristics of mice in the sham control (Contl) versus cecal ligation and puncture (CLP) groups administered *L. rhamnosus* fa1 (fa1), *L. rhamnosus* fg2 (fg2), or vehicle control (PBS; Veh) at 24 h post-operation, including: the representative fluorescent staining images of tight junction molecules, including occludin-1, claudin-1, and zona-occludens-1 (ZO-1) with the fluorescent intensity (**A**–**D**), gene expression of occluding-1 and ZO-1 in the colon tissue (**E**, **F**), gut permeability defect (FITC-dextran) (**G**), bacteremia (H), endotoxemia (I), serum cell-free DNA (**J**), and serum cytokines (TNF-α, IL-6, and IL-10) (**K**–**M**), are demonstrated (*n* = 6 per group). Data shown are pooled from 3 replicated experiments. *p < 0.05 between the indicated groups; ^#^*p* < 0.05 vs. control (Contl). The comparison was determined by the Student’s t test and one-way analysis of variance followed by Tukey’s analysis.

**Fig. 6:**
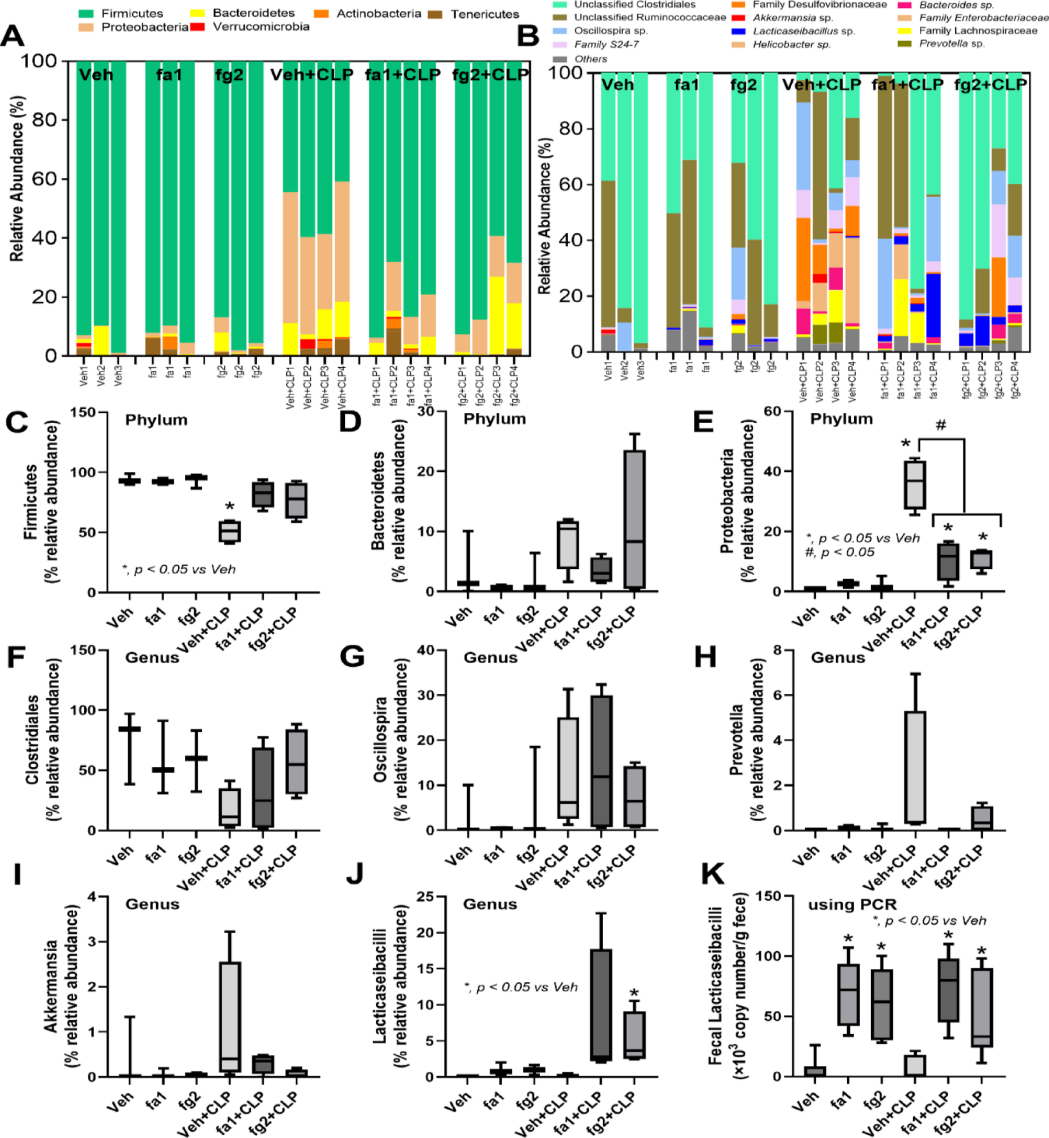
Fecal microbiome analysis of mice in the sham control (Contl), versus cecal ligation and puncture (CLP) groups after the administration by *L. rhamnosus* fa1 (fa1), *L. rhamnosus* fg2 (fg2), or vehicle control (PBS; Veh) at 24 h post-operation, as indicated by fecal relative abundance of bacteria in the phylum level (**A**) and family or genus (**B**) with the selected graph presentation of the abundance of bacteria in the phylum level (Firmicutes, Bacteroidetes, and Proteobacteria) (**C**–**E**), and in the genus level (*Clostridiales*,* Oscillospira*,* Prevotella*,* Akkermansia*, and *Lacticaseibacilli*) (**F**–**J**), are demonstrated. Also, the fecal abundance of *Lacticaseibacilli* using polymerase chain reaction (PCR) (K) are also shown (*n* = 3 for Veh, fa1, and fg2, while *n* = 4 for Veh + CLP, fa1 + CLP and fg2 + CLP). *p < 0.05 between the indicated groups; ^#^*p* < 0.05 vs. control (Contl). The comparison was determined by the Student’s t test and one-way analysis of variance followed by Tukey’s analysis.

Probiotics, even among the same bacterial species, can exhibit different properties^53^. Here, we used two strains of *L. rhamnosus*, fa1 and fg2, that we previously isolated and found to have differences in SCFA-producing properties; fg2 produces only acetate, while fa1 synthesizes acetate, butyrate, and propionate (Fig. 2A–C). Because of the well-known impact of probiotics on sepsis^8,9,12,13^, administration of fa1 and fg2 might attenuate sepsis, which, at least in part, is due to the elevation of fecal SCFAs. Then, we tested the impacts of probiotics in the CLP sepsis model. Accordingly, administration of probiotics to CLP sepsis mice had various effects, including improving their survival rates; reducing multi-organ damage to the kidney (serum creatinine and renal histology), liver (alanine transaminase), lung (histological score), and spleen (apoptosis) (Figs. 3A–H and 4A). In the lung injury, neutrophil infiltration in the lung of sepsis mice was demonstrated by the hematoxylin and eosin (H&E) strain, which was more prominent in CLP group without probiotic treatment (Fig. 3H, left side). Notably, renal histological injury in our model was mainly demonstrated through the loss of renal tubular cells but not immune cell infiltration, as indicated by the H&E stain (Fig. 4A, left side). However, there was no renal fibrosis in the kidney at 24 h post-CLP surgery compared with the control, as evaluated by Masson’s trichrome color (Fig. 4A, right side). Because SCFAs produced from probiotics might be associated with the intestinal alteration, fecal and serum SCFAs were determined. Interestingly, both probiotics similarly elevated SCFAs (acetate, butyrate, and propionate) in either feces or serum (Fig. 4B-D), despite the more prominent SCFAs production of fa1 over fg2 in vitro (Fig. 2A-C). Perhaps, both probiotics facilitated the growth of SCFAs-producing bacteria in mice, as previously mentioned^54^. Due to the well-known benefits of SCFAs toward enterocytes^55^, the intestinal injury was further evaluated. Indeed, both probiotics similarly restored enterocyte tight junction molecules (occludin-1, claudin-1, and ZO-1) (Fig. 5A–D), elevated expression of *Occludin* and *ZO-1* (Fig. 5E, F), and improved gut permeability (FITC-dextran assay) (Fig. 5G). The strengthening of gut permeability by probiotics partly resulted in a reduction of the translocation of bacteria, lipopolysaccharide (LPS), and cell-free DNA from the feces to the blood circulation, as indicated by a decrease in these parameters in probiotic-treated sepsis mice (Fig. 5H–J). Unsurprisingly, serum cytokines (TNF-α, IL-6, and IL-10) in probiotic-treated CLP sepsis mice were lower than in the untreated CLP group (Fig. 5K–M). However, attenuation of these sepsis parameters was surprisingly similar between mice treated with fa1 and fg2 probiotics (Figs. 3, 4 and 5), despite their different SCFAs production properties (Fig. 2A–C). Moreover, fecal microbiome analysis and fecal abundance of probiotics were performed (Fig. 6A–K). As such, sepsis-induced dysbiosis in CLP mice (Veh + CLP) was demonstrated by reduced Firmicutes (mostly Gram-positive anaerobes with health benefits) with increased Proteobacteria (a group of some Gram-negative pathogenic aerobes) without an altered Bacteroidetes (Fig. 6A–J). The abundance of fecal *Lacticaseibacilli* in control mice with probiotics (fa1 or fg2) was higher than in the control mice without probiotics, as determined by PCR but not by microbiome analysis (Fig. 6J, K). These data support the technical differences between 16 S rRNA sequencing and the more specific primer using PCR^38^. Despite the different SCFAs-producing properties between fa1 and fg2 (Fig. 2A–C), both probiotics normalized Firmicutes and reduced the abundance of Proteobacteria (Fig. 6A-E). Notably, administration of fg2 in CLP mice (fg2 + CLP), but not fa1 in CLP mice (fa1 + CLP), significantly increased fecal *Lacticaseibacilli* when compared with control (Fig. 6J), while the elevated abundance of fa1 and fg2 in CLP mice was similar by PCR from fecal samples (Fig. 6K). These data support the technical differences between microbiome and PCR^38^. The similar fecal microbiome alteration after the administration of fa1 and fg2 (the probiotics with different SCFAs-producing activity) implied other beneficial factors of probiotics that are not correlated with SCFAs; for example, anti-oxidants, exopolysaccharides, and nutrients competing with other bacteria^33^.

### Enhanced butyrate production, one of the underlying mechanisms of sepsis Attenuation by probiotics

**Fig. 7:**
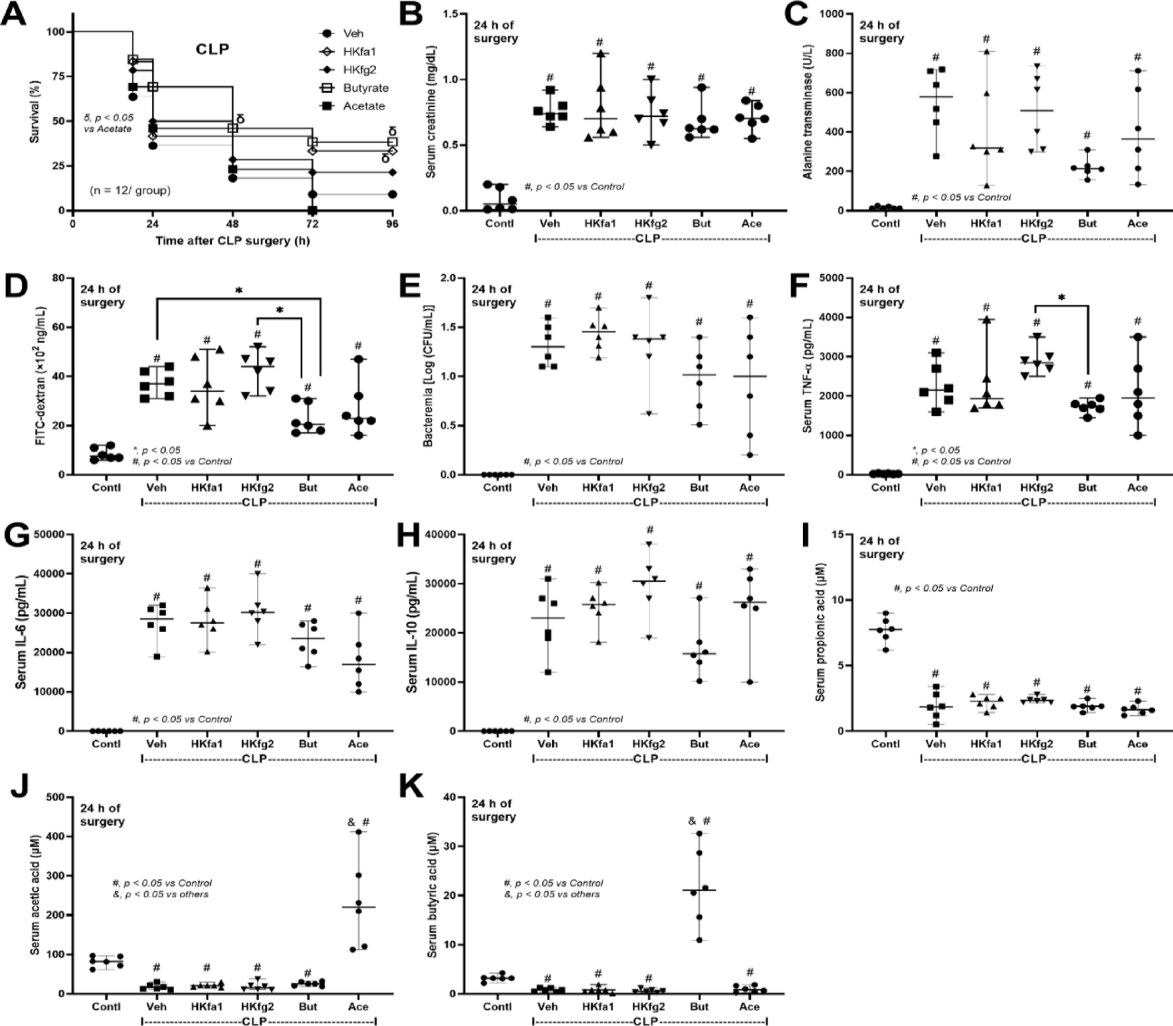
Characteristics of mice in the sham control (Contl) versus cecal ligation and puncture (CLP) groups administered with normal saline as a vehicle control (Veh) or heat-killed *L. rhamnosus* fa1 (fa1) and fg2 (fg2) or butyrate or acetate at 24 h post-operation, including: survival (**A**), renal function (serum creatinine) (**B**), liver injury (alanine transaminase) (**C**), gut permeability defect (FITC-dextran) (**D**), bacteremia (**E**), serum cytokines (TNF-α, IL-6, and IL-10) (F–H), and serum short-chain fatty acid (propionic, acetic, and butyric acid) (I-K), are demonstrated (*n* = 6 per group). *p < 0.05 between the indicated groups; ^#^*p* < 0.05 vs. control (Contl); ^&^*p* < 0.05 vs. other groups. The survival analysis was calculated by the log-rank test, while other comparisons were determined by the Student’s t test and one-way analysis of variance followed by Tukey’s analysis.

**Fig. 8:**
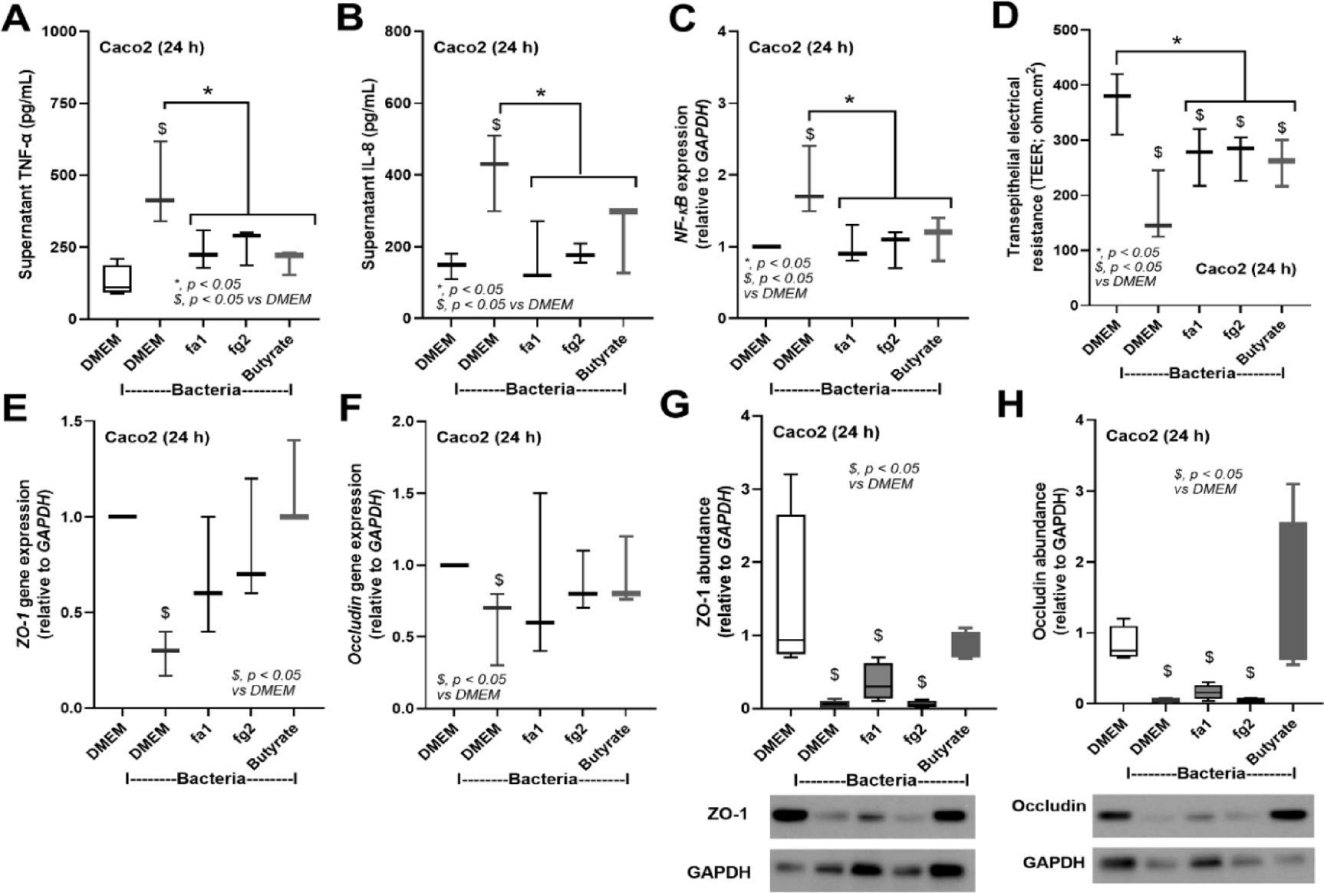
Characteristics of human enterocytes (Caco-2 cell line) after 24 h activation by culture media control (DMEM) or bacterial *K. pneumoniae* preparation (Bacteria; see methods), with DMEM or conditioned media from *L. rhamnosus* fa1 (fa1) or *L. rhamnosus* fg2 (fg2) or butyrate, including: supernatant cytokine (TNF-α and IL-8) levels (**A**, **B**); gene expression levels of *NF-κB* (**C**), and transepithelial electrical resistance (TEER) (**D**), and the tight junction molecules (occludin, and ZO-1) in the gene expression (polymerase chain reaction) (**E**, **F**) and the protein level with the representative picture of Western blot analysis (**G**, **H**), are demonstrated (independent experiments were performed in triplicate). *p < 0.05 between the indicated groups; ^$^*p* < 0.05 vs. DMEM. The comparison was determined by the Student’s t test and one-way analysis of variance followed by Tukey’s analysis.

We hypothesized that (i) SCFAs (butyrate or acetate) produced from the probiotics themselves or from other bacteria might partly be an underlying mechanism of sepsis attenuation by probiotics, and (ii) the viable probiotics were necessary for the communication with other gut organisms. Then, several factors (butyrate, acetate, and heat-killed probiotics) were treated in the CLP model. For survival analysis, there was no difference between all treatments and untreated CLP; however, the treatment with butyrate and heat-killed fa1 demonstrated better survival than acetate treatment (Fig. 7A). While heat-killed probiotics and acetate did not attenuate any 24 h post-sepsis parameters (kidney and liver injuries, leaky gut, and serum cytokines), butyrate attenuated some parameters, including liver injury (alanine transaminase), gut permeability (FITC-dextran), and serum TNF-α (no effect on serum IL-6 and IL-10) (Fig. 7B–H). These data implied the importance of butyrate on sepsis attenuation. Notably, the administration of butyrate and acetate elevated butyrate and acetate in serum, respectively, without an alteration in propionate (Fig. 7I-K). As probiotics and butyrate can produce factors that might directly protect enterocytes^56–58^, we next tested the effects of probiotic supernatants or butyrate on the enterocytic cell line (Caco-2). As such, the treatment of Caco-2 cells with Gram-negative bacteria led to cell damage, evidenced by elevated levels of cytokines (TNF-α and IL-8) in the cell supernatant and increased *NF-κB* (transcription factor) expression (Fig. 8A–C). In parallel, there was reduced tight junction molecules (occludin and ZO-1) (PCR and Western blot) and decreased transepithelial electrical resistance (TEER), relative to controls (Fig. 8D–H). Treatment with conditioned media from both probiotic strains and butyrate led to improvement of supernatant cytokines (TNF-α and IL-8), *NF-κB*, TEER, and the expression of *occludin* and *ZO-1* (Fig. 8A-F). Although probiotic supernatant and butyrate normalized *occludin* and *ZO-1* expression (Fig. 8E, F), only butyrate, but not probiotics, normalized occludin and ZO-1 in the protein level of detection (Fig. 8G, H and Supplement Figs. 2 and 3). Notably, the ratio between tight junction proteins/GAPDH is used to normalize the variability of GAPDH protein abundance. Again, no significant difference in the effects of conditioned media from the two probiotic strains was detected in vivo (Fig. 7A–K), despite their differences in SCFAs-producing properties in vitro (Fig. 2A–C).

### Reduced SCFAs in serum from patients with sepsis when compared with the healthy controls

**Fig. 9:**
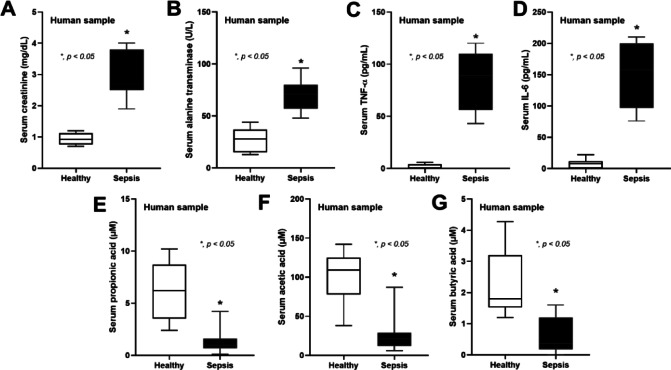
The characteristics of serum sample from the healthy control and patients with sepsis as indicated by serum creatinine (**A**), alanine transaminase (**B**), serum cytokines (TNF-α and IL-6) (**C**, **D**), and serum short-chain fatty acid (propionic, acetic, and butyric acid) (E-G) are demonstrated (*n* = 10 for heathy control and *n* = 12 for sepsis). *p < 0.05 between the indicated groups; ^$^*p* < 0.05 vs. DMEM. The comparison was determined by the Student’s t test and one-way analysis of variance followed by Tukey’s analysis.

Then, to emphasize the possible importance of SCFAs in patients with sepsis as a proof of concept, serum from healthy volunteers and patients (epidemiologic data presented in Table 1) was evaluated. Indeed, the higher serum creatinine, alanine transaminase, and cytokines (TNF-α and IL-6) with the lower SCFAs (propionate, acetate, and butyrate) in patients with sepsis compared with the healthy control was demonstrated (Fig. 9A–G).

**Table 1:** Demographic data of the participants.

	Sepsis (*n* = 12)	Healthy (*n* = 10)
Age, mean (SD)	43.7 (15.2)	37.6 (14.7)
Gender, male (%)	7 (58.3.7)	4 (40)
ICU days, median (IQR)	8 (7–15)	N/A
Hospital days, median (IQR)	18 (11–24)	N/A
SOFA score	8 (4–9)	N/A
Hemoglobin (g/dL), mean (SD)	7.90 (1.21)	13.05 (2.10)
WBC count (×10^9^/L), mean (SD)	13.23 (7.22)	5.78 (1.67)
Absolute neutrophil count (×10^9^/L), mean (SD)	9.72 (5.53)	3.15 (1.14)
Absolute lymphocyte count (×10^9^/L), mean (SD)	1.76 (0.85)	1.94 (0.78)
Respiratory infection (%)	7 (58)	N/A
Abdominal infection (%)	5 (42)	N/A
Others (%)	2 (17)	N/A

SD; standard deviation, IQR; internal quartile range, ICU; intensive care unit, SOFA; The Sequential Organ Failure Assessment, WBC; white blood cells, N/A; not applicable.

## Discussion

Despite notable improvements in reducing sepsis mortality rates, sepsis remains a significant contributor to global mortality. Sepsis is a common syndrome that develops in response to severe infection and leads to changes in host metabolites. In a CLP sepsis mouse model, metabolome analysis revealed higher levels of acetate and 3-hydroxybutyrate in the blood, and lower levels of butyrate and propionate in feces compared to sham controls. Administering fa1 or fg2 probiotics, but not heat-killed probiotics, before surgery effectively mitigated sepsis severity in mice.

In animals, fatty acids (FAs) can be derived from pyruvate, a byproduct of carbohydrates (glycolysis) and protein metabolism, and received from dietary sources (red apples, cheese, dark beer, and red wine)^59^. Also, FAs are normally synthesized in the liver, adipose tissue, and lactating mammary glands^60^. Dietary FAs, particularly long-chain (≥ 12 carbons) and medium-chain (6–12 carbons) FAs, primarily comprise triglycerides derived from animal fats and plant oils. In contrast, SCFAs are the end products of dietary fiber and non-digestible carbohydrate fermentation by gut microbes^60^. Free fatty acids (FFAs) are important energy sources, and the compromise of β-oxidation (a FFA breakdown pathway) due to sepsis-induced liver injury causes the accumulation of FFAs in the blood and organs^36^. Here, in comparison with control mice, acetate and 3-hydroxybutyrate were elevated in the blood of sepsis mice, while acetate in feces was not changed and fecal 3-hydroxybutyrate was lower in sepsis. Because gut microbial SCFAs play a role in sepsis by translocating to the bloodstream^15^, the lower or unchanged levels of SCFAs in feces but higher SCFAs in serum imply the translocation of SCFAs from the gut into the blood circulation during sepsis. The molecules of SCFAs are small enough to pass through the intact tight junction (TJ) of enterocytes, which allows the translocation of molecules smaller than 600 Da^9^(molecular weight of acetic acid 60.05 Da, propionic acid 74.08 Da, and butyric acid 88.11 Da). With sepsis-induced TJ damage, the translocation of SCFAs might be facilitated. Also, the depleted butyrate and propionate in the feces of sepsis mice might be due to the reduced abundance of Firmicutes bacteria in CLP mice, as described in our previous publications^13,61^. Although enteric bacteria generate acetate, propionate, and butyrate in a 3:1:1 stoichiometry, which can be taken up through the colon, most of the elevated blood acetate in sepsis is attributable to de novo production by pyruvate-derived acetate overflow, protein deacetylation, and acetyl-CoA hydrolase^38,62^. Hence, the elevation in acetate, β-hydroxybutyrate (a ketone body), lactate (anaerobic by-products), and pyruvate in mouse blood support reduced acetyl-CoA due to enhancement of alternative metabolic sources during sepsis (a nutritionally depleted condition)^36,63^. The reduction in alanine (a branched-chain amino acid) alongside increased pyruvate in sepsis model may be due to alanine transformation into pyruvate for subsequent energy production via entry into the tricyclic acid cycle^36^. Furthermore, the reduction in SCFAs (butyrate and propionate) with altered amino acid levels in the feces of sepsis mice support the occurrence of sepsis-induced gut dysbiosis that is, at least in part, responsible for increased sepsis severity^37,64^.

**Fig. 10:**
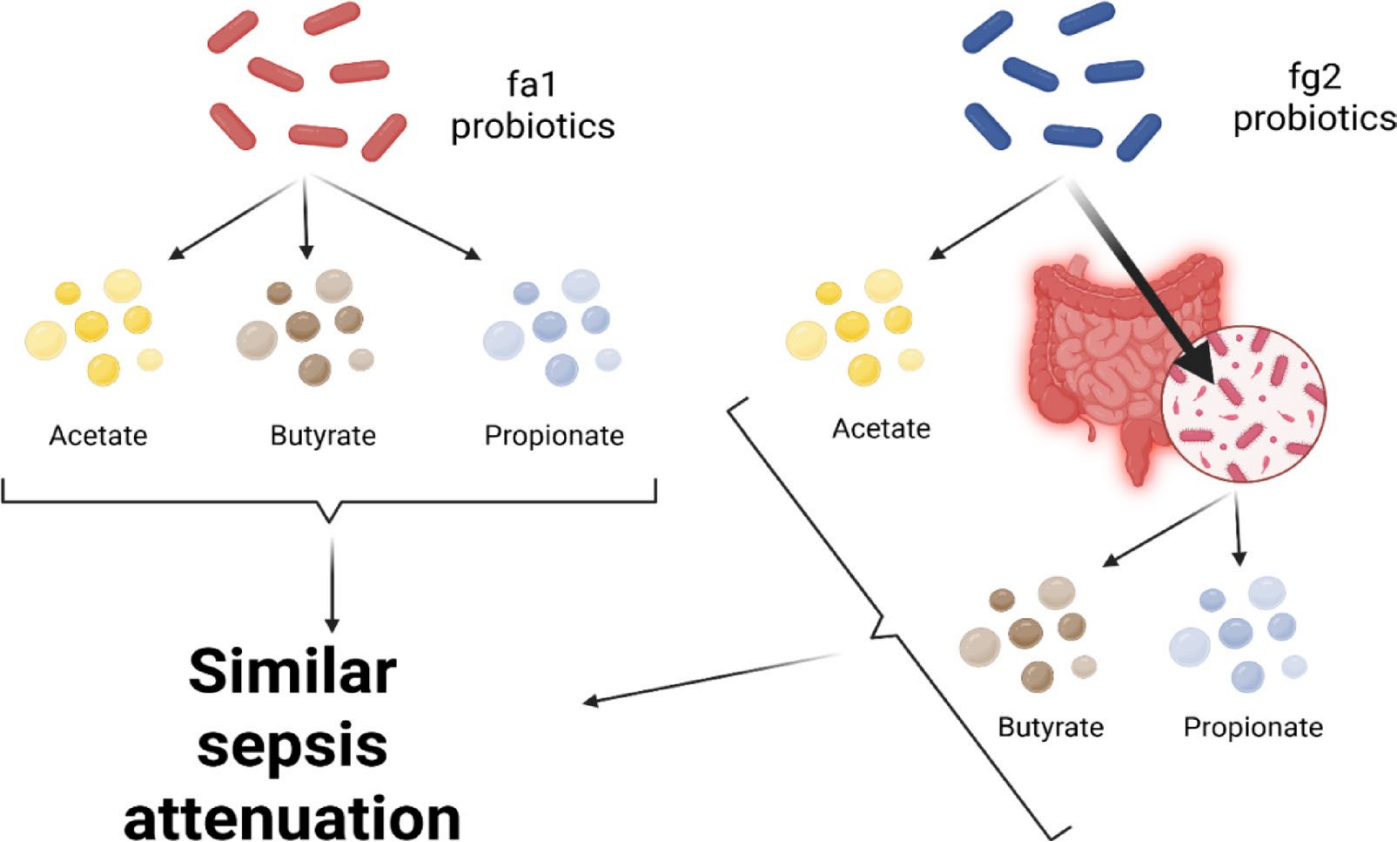
The proposed hypothesis demonstrates the possible different origin of short-chain fatty acids (SCFAs) between *L. rhamnosus* fa1 and fg2 probiotic strains. While fa1 and fg2 produce 3 SCFAs (acetate, butyrate, and propionate) and only acetate, fg2 demonstrates a property to induce some beneficial bacteria that could produce butyrate and propionate. Despite the possible different sources of SCFAs, both fa1 and fg2 similarly elevate SCFAs in mice with similar beneficial effects on sepsis attenuation.

Given the depletion of fecal SCFAs in our model and the enterocyte protection properties of SCFAs, treatment with probiotics that can alter SCFAs may improve the outcomes of the sepsis mice. Surprisingly, 1 week of administration of both strains of probiotic prior to sepsis induction provided similar protection to sepsis CLP mice, as demonstrated by survival analysis and several parameters at 24 h post-CLP, including organ damage (lung, liver, kidney, and spleen), leaky gut, serum cytokines, endotoxemia, and bacteremia, despite the difference in their SCFAs production properties. There was prominent production of SCFAs in fa1 strain (produced acetate, butyrate, and propionate) compared with fg2 probiotics (mainly produced acetate) (Fig. 2A–C). Interestingly, the administration of fa1 and fg2 similarly elevated SCFAs in both feces and serum of sepsis mice (Fig. 4B–D), despite the difference in SCFAs producing properties between fa1 and fg2. While the production of SCFAs from fa1 probiotics may be an important mechanism for sepsis attenuation, benefits of fg2 strain with limited production of SCFAs need more explanation. Interestingly, the elevated levels of butyrate and propionate in blood and feces of mice administered with fg2 (the probiotics mainly produced acetate in vitro), implying the importance of butyrate and propionate produced from other organisms in the mouse gut (in vivo) (Fig. 10). Indeed, the overgrowth of beneficial bacteria in feces after probiotic administration is previously mentioned^65^. Despite uncertain sources of butyrate and propionate after the administration of fg2, fg2 attenuated sepsis, and the acetate-producing property of fg2 alone could not explain the benefit of fg2 in sepsis because acetate administration did not attenuate sepsis in mice. More studies are interesting.

These data implied the importance of the induction of SCFAs production from other gut bacteria after *L. rhamnosus* administration, while the SCFAs production property of the probiotics themselves seems to be a less important factor to predict the benefit in sepsis. Meanwhile, the communication between probiotics and other microbes in the gut was important, supporting by the loss of effectiveness in the heat-killed probiotics. The viability of probiotics after administration in the feces of sepsis mice at 24 h after CLP was also supported through microbiome analysis and fecal PCR (Fig. 6J, K). While probiotic administration increased *Lacticaseibacilli* spp. in CLP mice as determined by both microbiome and PCR, *Lacticaseibacilli* spp. in probiotic-administered control mice was detected by PCR but not by microbiome analysis (Fig. 6J, K). Indeed, the 16 S rRNA sequencing in microbiome analysis might not detect all bacteria present, especially at the low abundance, compared with the use of PCR with more specific primers^38^. Based on our microbiome analysis, *Lacticaseibacilli* spp. might be more prominent in sepsis than in control non-sepsis mice. Perhaps normal microbiota in the healthy mice reduce the abundance of probiotics (an ability to control foreign organisms), and the microbial control ability is impaired in sepsis-induced dysbiosis^21,22^. More studies are interesting. Nevertheless, both probiotics similarly increased Firmicutes (mostly beneficial Gram-positive anaerobes) and reduced Proteobacteria (a group of some pathogenic Gram-negative aerobes), despite the different SCFAs-producing properties. Interestingly, the administration of heat-killed probiotics did not elevate SCFAs in feces and serum, emphasizing the production of SCFAs from other microbes in the gut. Hence, other beneficial bacteria in the gut might be important for the impact of probiotics on sepsis attenuation^65^ and also demonstrated by the use of fecal transplantation^66^. Further studies using germ-free mice might be interesting.

Among several SCFAs, acetic acid is the main SCFAs produced by most beneficial microbiota in the human gut^67^ and confers health benefits via changes in gut bacteria^51,68,69^, including improvements in enterocyte function^70^ and increased IgA responses^71,72^. Despite the benefits of both fa1 and fg2 probiotics, which could similarly produce acetic acid, administration of acetate did not attenuate sepsis severity (Fig. 7A-H). Meanwhile, butyrate, another important SCFAs, attenuated some aspects of sepsis, including serum TNF-α and leaky gut. However, other additional non-SCFAs beneficial factors of probiotics; for example, other anti-inflammatory molecules^33^ and exopolysaccharides^73–76^ might be important. Likewise, the interference in several metabolic profiles in the gut (bile acid, lysophosphatidylcholines, and eicosatetraenoic acid^77^, or a mixture of SCFAs^78^, and butyrate concentrations^79^, might be responsible for the better effect of probiotics over butyrate in our study. Notably, the serum level of butyrate from the direct butyrate administration (Fig. 7K) was approximately 5 folds higher than the levels of butyrate from the probiotic induction (Fig. 4D). Hence, one of the mechanisms of sepsis attenuation from our probiotics^80,81^ might be an elevation of butyrate. Moreover, the repeated administration of *L. rhamnosus* might be necessary to maintain the beneficial impacts of probiotics, due to the loss of bacteria within 3–5 days after stopping administration (non-colonization)^38^. Duration of the increased butyrate, as a cross-species metabolic pathway (an elevated butyrate production from other bacteria)^82^, after 24 h of the administration, need further tests in other models. Because SCFAs rely on the fermentation of carbohydrate or non-human digestible fiber^83^, the production of SCFAs in standard culture media used only a limited amount of carbohydrate^67^. Then, the production patterns of SCFAs of fa1 and fg2 in the enhanced environment (prebiotics)^84^ might be different from the current results. Here, culture media from the probiotics (butyrate approximately at 1 mM; Fig. 2B) protected enterocytic pro-inflammation induced by bacterial lysate (TEER, *ZO-1* and *occludin* gene expression, but not in the protein levels). Meanwhile, butyrate in a higher concentration (2 mM) attenuated inflammation and improved the tight junction proteins both in the gene and protein levels. Thus, our in vitro data also highlighted the importance of butyrate on enterocyte protection. For clinical translation, our data support the use of probiotics for sepsis prevention without the necessity to measure the production of SCFAs in vitro. Also, SCFAs in feces and serum might be the interesting biomarkers to determine the impacts of probiotics in sepsis.

Although an alteration of SCFAs and the benefits of probiotics in sepsis are previously mentioned^8,33^, there are a few novel findings from our data. First, the similar sepsis attenuation property of probiotics did not depend on the production of SCFA. The selection of probiotics to use for sepsis should also be based on the in vivo data. Different probiotic strains employ diverse and specific mechanisms of action, some of which may converge to produce similar beneficial outcomes in the host^85^. Here, the mechanisms by which fg2 (acetate-producing probiotics) induced butyrate and propionate from other gut bacteria (Fig. 10) were interesting. Second, the viability of probiotics was important for the sepsis attenuation effect, at least for some probiotics. Third, the reduced levels of serum SCFAs in patients with sepsis might be an indication for probiotic use. More studies are warranted to answer these questions. Moreover, several limitations should also be mentioned. First, only abdominal sepsis from CLP surgery was tested with only the limited number of mice. The sepsis from other causes, for example, pneumonia, skin infection, and urosepsis, might demonstrate different conclusions. Then, the conclusions were still preliminary. Second, the underlying mechanisms of action between fa1 and fg1 (the probiotics from same species) were not deeply explored. Third, the tests of SCFAs and probiotics as sepsis biomarkers and treatment strategy, respectively, were not performed in patients. More studies are interesting.

### Clinical perspectives

- Both probiotics with the prominent SCFAs (providing butyrate, propionate, and acetate) (fa1) or the limited SCFAs (mainly providing acetate) (fg1) similarly attenuated sepsis severity with similar level of beneficial SCFAs in feces and in serum.
- The effectiveness of probiotics was loss after heat-killed procedure supporting the importance of bacterial communication to induce the production of SCFAs from other bacteria in the gut.

## Supplementary Information

Below is the link to the electronic supplementary material.

Supplementary Material 1

Supplementary Material 2

Supplementary Material 3

## Data Availability

The datasets generated during and/or analyzed during the current study are available from the corresponding authors on reasonable request.
