# Supplementary figures and images for: Modulation of sepsis by Lacticaseibacillus rhamnosus and the potential role of short-chain fatty acid levels in feces and blood
Source: Sci Rep. 2025 Dec 24;16:3102. doi: 10.1038/s41598-025-33032-4 (PMC12830833; doi:10.1038/s41598-025-33032-4)

**Supplement Figure 3.** The representative Western Blot analysis of Figure 8H.

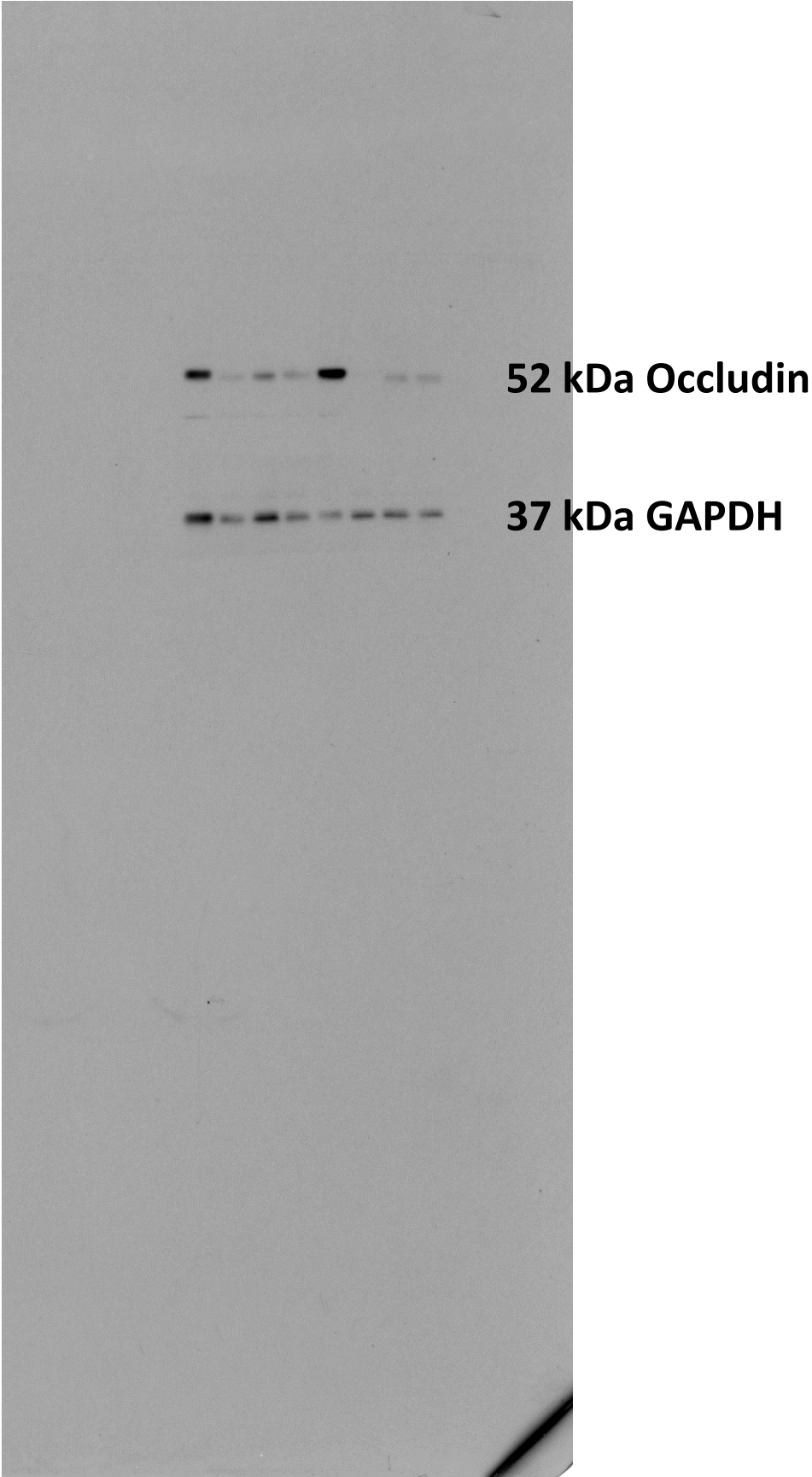

Supplement: Supplementary file 1 — Supplementary Material 1 [file 41598_2025_33032_MOESM1_ESM.pdf]

**Supplement Figure 2.** The representative Western Blot analysis of Figure 8G.

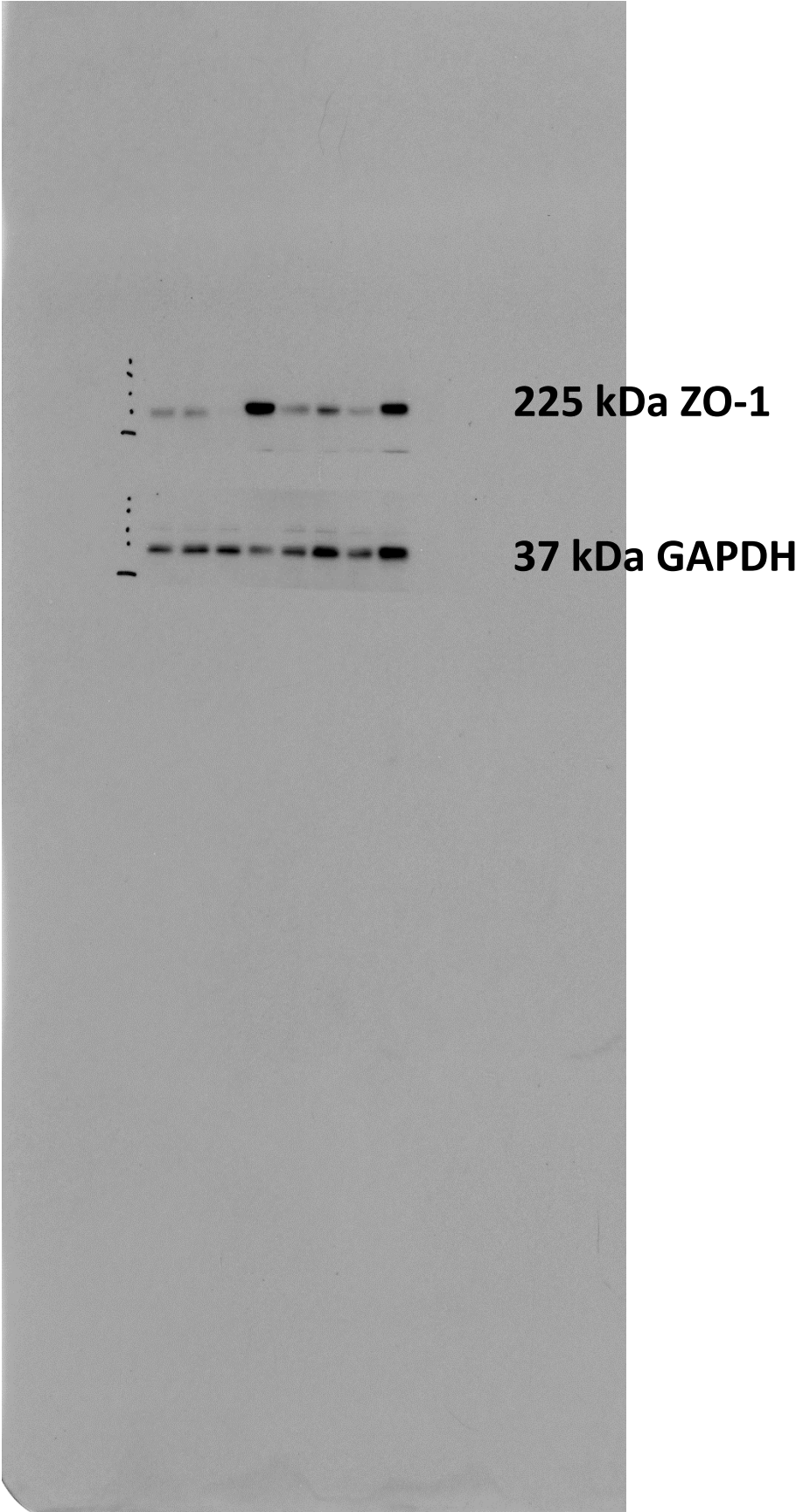

Supplement: Supplementary file 2 — Supplementary Material 2 [file 41598_2025_33032_MOESM2_ESM.pdf]
