# Supplementary material for: Modulation of sepsis by Lacticaseibacillus rhamnosus and the potential role of short-chain fatty acid levels in feces and blood
Source: Sci Rep. 2025 Dec 24;16:3102. doi: 10.1038/s41598-025-33032-4 (PMC12830833; doi:10.1038/s41598-025-33032-4)
Supplement: Supplementary file 3 — Supplementary Material 3 [file 41598_2025_33032_MOESM3_ESM.pdf]

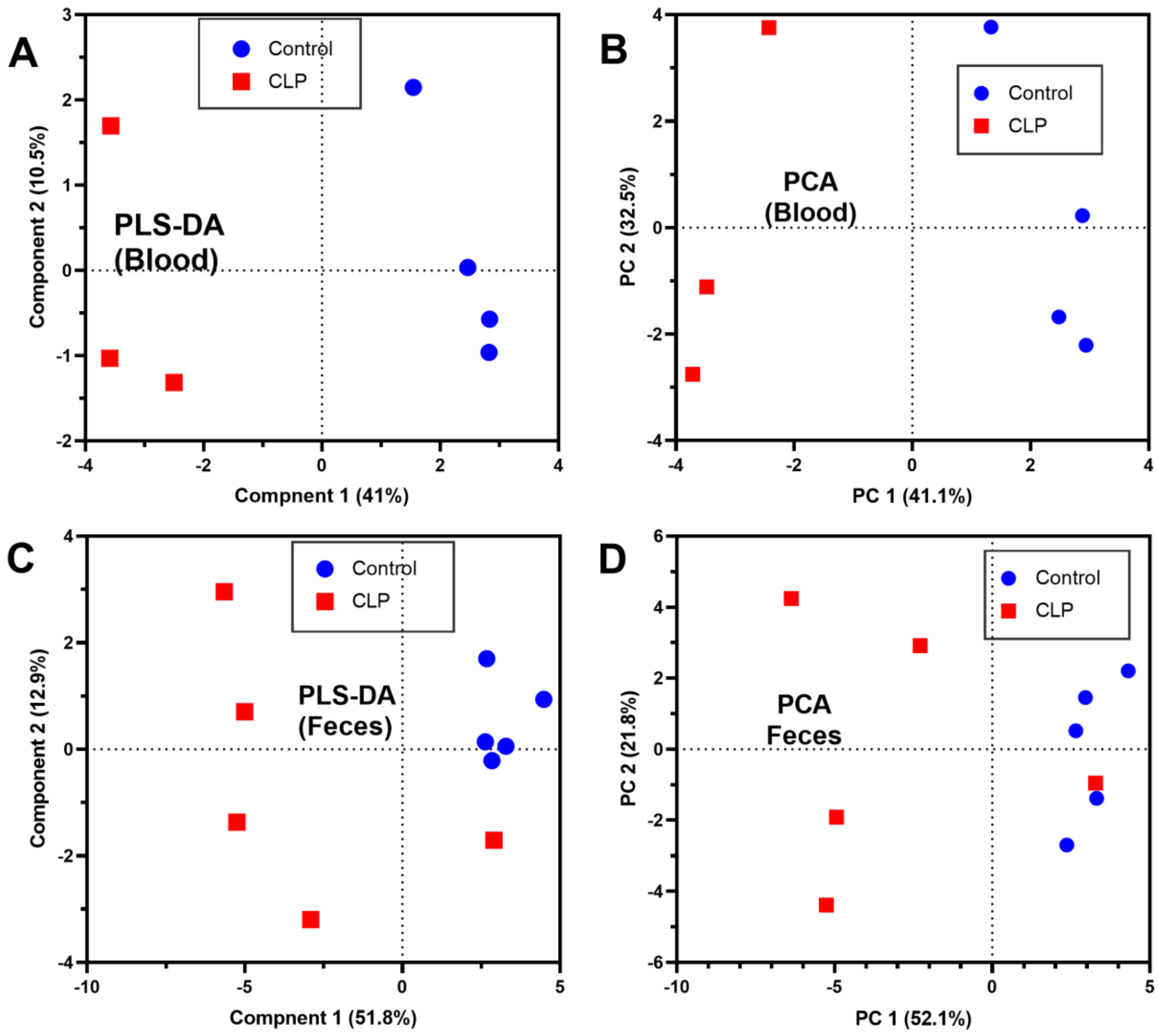


**Supplement Figure 1.** Score plot analyses of the relationships among metabolome profiles to reduce the dimensionality of multivariate data into a clearer structure, as generated by principal-components analysis is partial least squares-discriminant analysis (PLS-DA; using the first 2 latent variables) and principal component analysis (PCA; using all variables) of mice with at 24 h post-operation of sham (control) versus cecal ligation and puncture (CLP) from the blood samples (A, B) (n = 4 for control and n = 3 for CLP in the blood metabolome) and the fecal samples (C, D) (n = 5 per group in the fecal metabolome) are demonstrated.
